# Case report: Restrictive cardiomyopathy presenting with complete thromboembolism occlusion of the terminal part of the abdominal aorta in a preadolescent Saudi girl

**DOI:** 10.3389/fped.2022.944627

**Published:** 2022-07-19

**Authors:** Ahmad A. Al-Shammari, Rawan Al Muslim, Jenan Almuslim, Ehab Elashaal, Haitham Lardhi, Saleh A. AlQahtani, Bassam N. AlBassam, Amer Lardhi

**Affiliations:** ^1^Department of Pediatrics, College of Medicine, Imam Abdulrahman Bin Faisal University, Dammam, Saudi Arabia; ^2^King Fahd Hospital of the University, Khobar, Saudi Arabia; ^3^College of Medicine, Imam Abdulrahman Bin Faisal University, Dammam, Saudi Arabia; ^4^Department of Surgery, College of Medicine, Imam Abdulrahman Bin Faisal University, Dammam, Saudi Arabia

**Keywords:** restrictive cardiomyopathy (RCM), thromboembolism, abdominal aorta, cardiac transplant, pediatrics, Saudi Arabia

## Abstract

Restrictive cardiomyopathy (RCM) is a rare disease in children, accounting for <5% of all pediatric cardiomyopathies. It may be idiopathic or may be a secondary to a systemic disease. The disease is characterized by normal systolic function with impaired ventricular filling caused by stiff ventricular walls. Children with RCM often present with symptoms of exercise intolerance, shortness of breath, weakness, and chest discomfort. Thromboembolism events are an unusual presentation of RCM. We are reporting a preadolescent female from the eastern province of Saudi Arabia who presented with sudden right lower limb swelling, paresthesia, and pain caused by a complete occlusion of the terminal part of the abdominal aorta and both common iliac arteries. Echocardiography revealed dilated atria, normal ventricle dimensions and two floating thrombi in the left atrium. The patient was successfully managed with an anticoagulant, surgical thrombectomy and cardiac transplantation.

## Introduction

Restrictive cardiomyopathy (RCM) is a rare condition in the pediatric age group which accounts for 2–5% of all pediatric cardiomyopathies. It may be idiopathic or may be associated with a systemic infiltrative disease, inborn errors of metabolism, malignancy, radiation therapy, or drug toxicity. Idiopathic RCM with no obvious identifiable cause is the most common form of the disease (1). RCM is characterized by severe diastolic dysfunction with almost preserved systolic function, and dilated atria

with normal or increased ventricular wall thickness (2). RCM has a poor prognosis, most patients die because of protracted heart failure, progressive pulmonary hypertension, and cardiac arrhythmia (3, 4). Thromboembolic events can be a source of mortality or severe morbidity (5). Cardiac transplantation is the only effective treatment (7). In this article, we present a Saudi female child with lower limb pain and edema caused by thromboembolism of the terminal abdominal aorta as a manifestation of idiopathic RCM.

## Case description

An 11-year-old Saudi girl presented to the emergency room with sudden right lower limb swelling, paresthesia, and pain for 1-day duration. The patient reported no history of shortness of breath, chest pain, palpitations, or syncopal attacks. General physical examination showed an active, alert, afebrile preadolescent girl with no signs of respiratory distress. She was unable to stand and walk. Vital signs include Temperature: 36.5°C, Blood pressure: 100/70 mmHg, Heart rate: 82 beats/minute, Respiratory rate: 20 breaths/minute, Oxygen saturation: 97% breathing room air. Growth parameters were as follows: weight, 47 kg on the twenty fifth centile for age and sex; height, 154 cm between the twenty fifth and fifty centile for age and sex. Pertinent cardiac examination showed a raised jugular vein. Precordium auscultation revealed normal first and second heart sounds with no added sound or murmur. Lung auscultation revealed vesicular breathing with equal air entry bilaterally and no added sounds. She had a soft and lax abdomen and just a palpable liver without tenderness or splenomegaly. Lower extremities examination showed pale and cold limbs with calf muscle tenderness. Pulses were absent up to the femoral artery of the right limb and up to the popliteal artery of the left limb. Tone, power, and deep tendon reflexes were normal.

**Figure 1:**
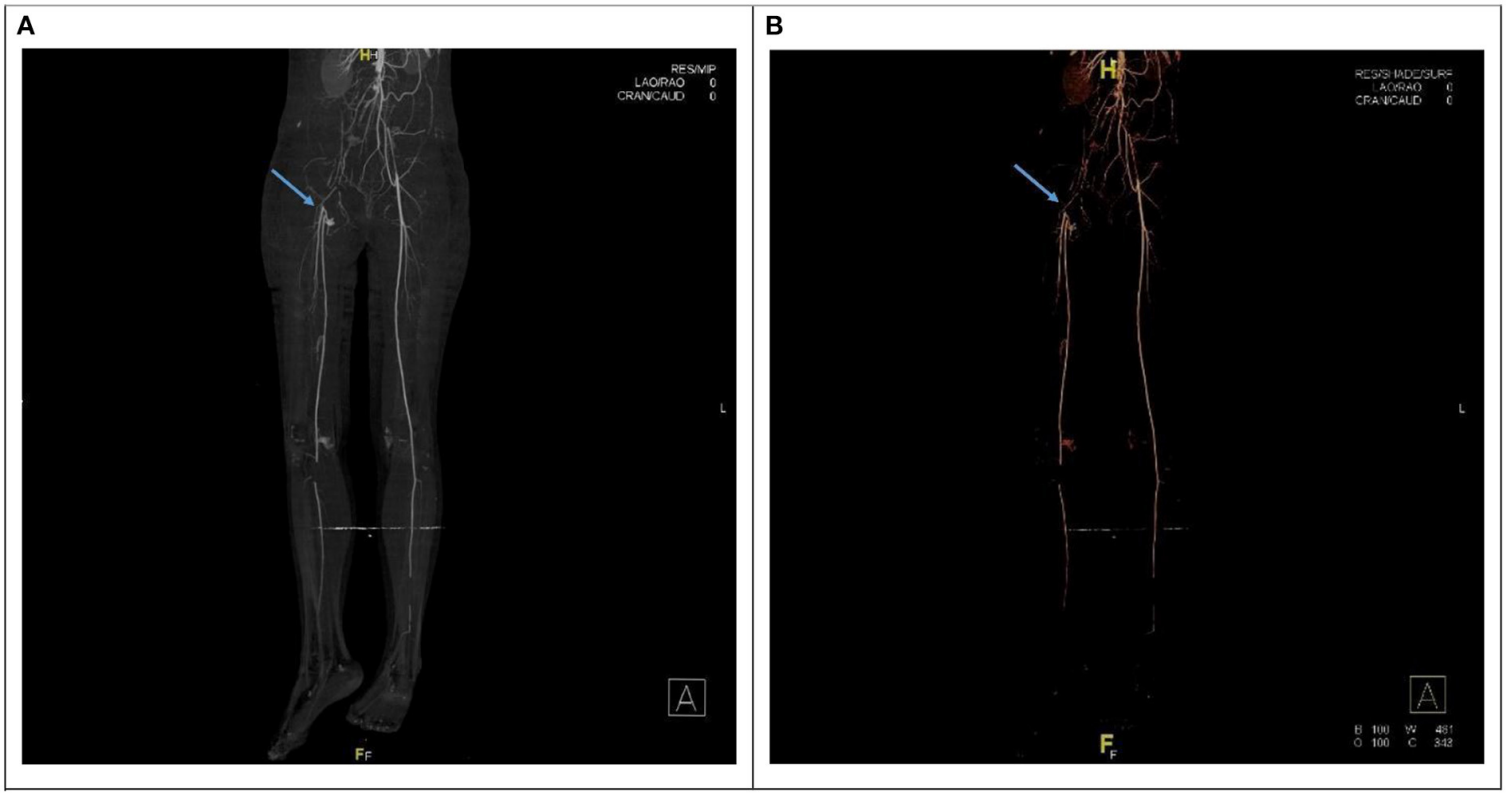
**(A,B)** CT angiogram showing interruption of the contrast at the aorto-iliac arterial level correlated with embolization at the aortic bifurcation (saddle embolus) with thrombus propagation. The blud arrow point to no visualization of the right external iliac artery.

**Figure 2:**
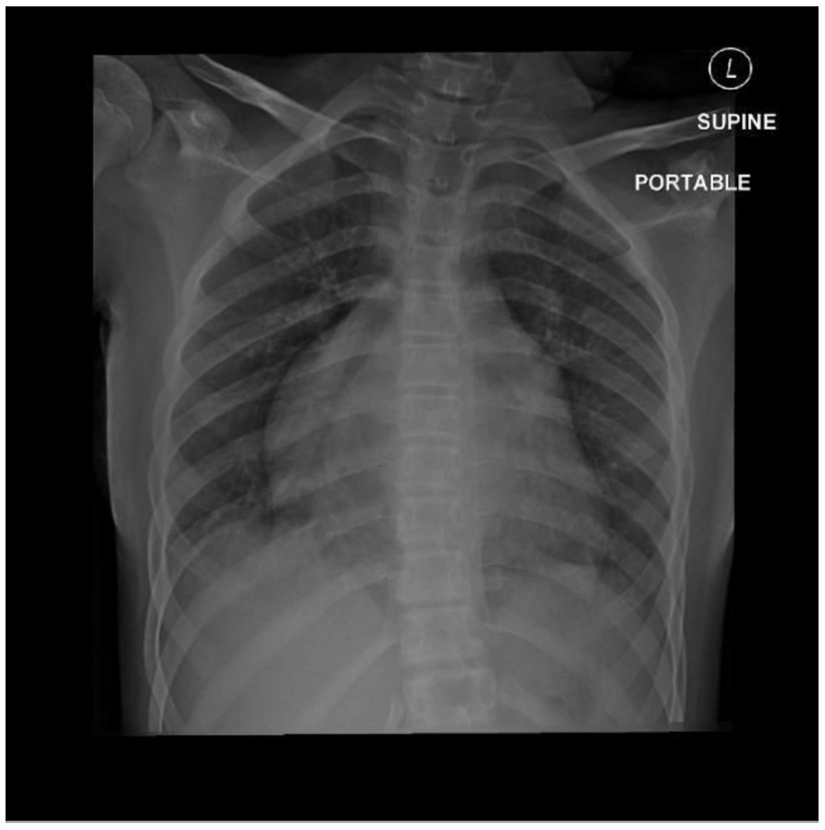
Chest x-ray showing cardiomegaly with a prominent atrium.

**Figure 3:**
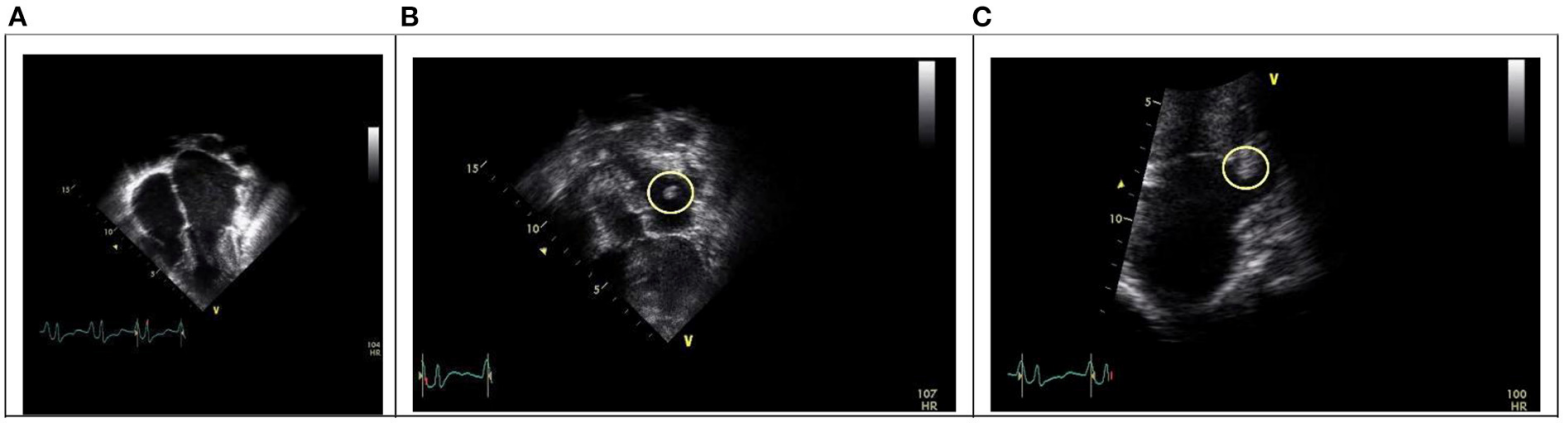
**(A)** Apical four chamber view showing bi-atrial dilatation with normal ventricles size. **(B)** A thrombus mass in the left atrium close to the left atrial appendage (yellow circle). **(C)** A thrombus mass in the left lower pulmonary vein.

Laboratory investigations showed normal complete blood count (CBC), creatine phosphokinase (CPK): 10,261 U/L (normal value 30–230 U/L), Myoglobin: 1,160 ng/ml (10–92 ng/ml), plasma D-dimer: 5.1 μg/ml (normal range, <1.0 mg/ml). Prothrombin time (PT) 15.6 seconds, partial thromboplastin time (PTT) 27.2 s (25–35). It was found to have normal Protein S, Protein C, Antithrombin III level. Also, her anti-beta2 glycoprotein antibody immunoglobulin M (IgM) and immunoglobulin G (IgG), Lupus anticoagulant, antinuclear antibody, and anticardiolipin IgM and IgG were negative. Radiological investigation included Doppler ultrasound of the lower limbs showed right femoral artery occlusion and left popliteal artery occlusion with patent veins. Computed Tomography Angiography (CTA) of bilateral lower extremity showed complete occlusion of the terminal part of the abdominal aorta and both common iliac arteries with no visualization of the right external iliac artery, the common femoral artery, and a short segment of the proximal popliteal artery (Figure 1). Chest X-ray showed cardiomegaly with a prominent left atrial wall (Figure 2). Electrocardiography showed bilateral atrial enlargement. Echocardiography revealed a hugely bi-atrial enlargement, two thrombus masses were seen in the left atrium close to the left atrial appendage and left lower pulmonary vein, normal sized ventricles with mildly hypertrophic walls, normal pericardium with Fractional shortening 42% (Figure 3). Such findings on echocardiography ruled out constrictive pericarditis in our patient. Furthermore, endocardial biopsy showed cardiomyopathic changes with interstitial fibrosis consistent for idiopathic RCM. No infiltrative or storage materials were detected in the mediastinal lymph nodes and endocardial biopsies. Genetic testing was not performed; however, no similar condition is reported from the patient's family.

The patient was started on intravenous Heparin infusion, Metoprolol 12.5 mg twice per day and Diuretics. After CT-angiography finding she underwent urgent bilateral lower limb and aortic embolectomy. However, her peripheral pulses remarkably improved after the procedure. She was maintained on Heparin which was replaced by Warfarin upon discharge along with metoprolol and diuretics. On follow-up, the patient developed arrhythmia, easy fatigability, and dyspnea in which she was admitted. However, due to ineffective medical management, she was listed for heart transplantation, and she underwent a successful transplant 11 months after onset of symptoms. She had a smooth post-transplantation course with resolution of cardiac symptoms.

## Discussion

The clinical presentation of RCM in children characterized by progressive symptoms of decreased cardiac output such as fatigue, dyspnea, and easy fatigability. While clinical examination can reveal heart failure signs such as increased jugular venous pressure, presence of third and fourth heart sound, pleural effusion associated finding (crackles or reduced air entry), hepatomegaly and lower limb edema (1). In addition to the clinical and radiological findings, diagnosis can be confirmed by echocardiography and endocardial biopsy (1, 2). However, diagnosis of RCM should be differentiated from hypertrophic cardiomyopathy and constrictive pericarditis (1).

There is no specific treatment for idiopathic RCM in comparison to certain causes of secondary RCM that may have specific therapies. However, the general management of patients with RCM include antithrombotic therapy, diuretics, and management of end-organ dysfunction. Diuretics doses are used and adjusted up on patient clinical status as restrictive physiology requires a good preload to maintain an adequate cardiac output (1, 6). In regards to antithrombotic therapy, the preferred treatment is aspirin or warfarin. It is recommended to be started at the time of diagnosis due to the risks of thrombus formation and embolization. There are no current studies comparing the effectiveness of preventing embolism between aspirin and warfarin in this subset of children. However, warfarin is hypothesized to be more effective in preventing embolism despite the concern of monitoring difficulties (6, 7). Decreasing ejection fraction by using beta blockers, angiotensin receptor blockers, and angiotensin converting enzyme inhibitors have not been shown to improve outcomes in adults or children. However, the use of beta blocker may be helpful in management of arrhythmia as in our patient or in those with ST segment depression and ischemia symptoms at higher abex beat (1, 7). Pacemakers or defibrillators therapy may be used in restrictive cardiomyopathy per standard guidelines in controlling rhythm. Cardiac transplant is the only effective treatment which should be promptly considered for children with previous embolic events diagnosed to have RCM (1, 6, 7). A regular monitoring by echocardiography for pulmonary vascular resistance is required to avoid the need of lung-heart transplantation instead of heart alone (1).

The course of RCM in children usually carries a very poor prognosis which is worsens if the patient experiences an embolism (6). The incidence of intracardiac thrombus in the literature ranges from 0 to 42% while the incidence of emboli elsewhere ranges from 12 to 33%. One of the major risk factors for embolism is atrial enlargement. Additionally, other factors include blood stasis and turbulent flow of the blood due to the distorted contours of a dilated atrium. In children with RCM, arrhythmia is common with an approximate incidence of 15%. Such arrhythmia may produce a greater blood turbulence and may promote a thrombus to embolize (6).

Pediatric free heart transplant survival rates of the 1–2–5 years are 48, 34, and 22% respectively. Pediatric heart transplant survival rates in the United Kingdom showed 96, 90, and 84% during the 30-days, 1-year and 5-years, respectively. However, transplanted RCM patients are generally much better than those on medical therapy only (1). Genetic testing screening for first family degree relatives plays a key role in identifying such a member at risk of developing the disease despite that prognostic value has not been clearly established (1). Additionally, it was noticed that cardiac transplant outcomes are similar among children with restrictive cardiomyopathy and other forms of pediatric heart disease (7).

In conclusion, RCM is a rare disease in children. It usually presents with symptoms of diastolic heart failure. The authors report a case of RCM with presenting symptoms of thromboembolic event. The case emphasizes the importance of considering RCM in children presenting with thromboembolic events.

## Data Availability Statement

The original contributions presented in the study are included in the article/Supplementary material, further inquiries can be directed to the corresponding author.

## Author Contributions

All authors listed have made a substantial, direct, and intellectual contribution to the work and approved it for publication.

## Conflict of interest

The authors declare that the research was conducted in the absence of any commercial or financial relationships that could be construed as a potential conflict of interest.

## Publisher's Note

All claims expressed in this article are solely those of the authors and do not necessarily represent those of their affiliated organizations, or those of the publisher, the editors and the reviewers. Any product that may be evaluated in this article, or claim that may be made by its manufacturer, is not guaranteed or endorsed by the publisher.

## Publisher's Note

All claims expressed in this article are solely those of the authors and do not necessarily represent those of their affiliated organizations, or those of the publisher, the editors and the reviewers. Any product that may be evaluated in this article, or claim that may be made by its manufacturer, is not guaranteed or endorsed by the publisher.
